# Supplementary material for: Methyl t-butyl ether-degrading bacteria for bioremediation and biocontrol purposes
Source: PLoS One. 2020 Feb 21;15(2):e0228936. doi: 10.1371/journal.pone.0228936 (PMC7034917; doi:10.1371/journal.pone.0228936)
Supplement: Attachment — Submitted filename: Response to Reviewers.doc [file pone.0228936.s001.doc]

16 December 2019

Dear Editor,

**MANUSCRIPT: PONE-D-19-29309**

We are very grateful to the editor and reviewers for their suggestions, which have been very helpful in improving the manuscript. The revised manuscript with track changes and an unmarked version have been uploaded as separate files. We have made a considerable effort to consider all the interesting suggestions and corrections proposed by the reviewers. However, should it be necessary, further comments and suggestions are welcome. Below, are the point-to-point answers to the comments of the reviewers.

On behalf of all co-authors,

Yours sincerely,

Francesco Vinale

Reviewer comments:

Reviewer #1:

1) Article it is interesting since identified bacterial strains putatively interesting in bioremediation of MtBE contaminated soils, but also analyzed their ability to induce growth and defense response in plants, which both are together very interesting properties. Thus, this work includes new insights in these two, apparently unrelated fields, bioremediation and biocontrol. The experiments are clearly presented and article well written.

*Thank you for your positive comments.*

2) Abstract (lines 22-23): Please specify if *B. aryabhattai* refers to a *Bacillus* or a *Bosea* species.

B. aryabhattai *refers to a* Bacillus *species. This information is now indicated in the text.*

3) Results: section starting on Line 277. It would be interesting to know why authors do not include strain *M. mucogenicum* R8i in this analysis. Please explain.

M. mucogenicum *R8i was not effective in reducing fungal activities. This information is now indicated in the text.*

4) Legend to Fig. 2 must be wrong since in that Figure there are included data from strains R1B, R8b and R8i, while in the legend indicates that only data from *B. aryabhattai* R1b are included. Please correct the legend.

*Legend has been corrected.*

5) Section starting on line 337. Authors would have to explain more in detail why they only used strain *M. mucogenicum* R8i in this study.

*We have not used only M. mucogenicum R8i. Data are shown in figure only for this bacteria because B. aryabhattai R1B and S. novella R8b did not induce a resistance in tomato plants as reported at lines 346-347.*

Reviewer #2:

The MS is overall well written, and authors presented using a multidisciplinary approach novelty results, reporting for the first time the occurrence of bioremediation and biocontrol activities of some species of Bacteria.

*Thank you for your positive comments.*

I suggest, if the authors had some good pictures of the experiment *in vivo*, to insert them in the MS or in the Supplementary Materials.

*We have some pictures of the experiment* in vivo *but the quality is not high. If it is necessary we could insert them as supplementary materials.*

If know, what is the effect of these selected bacteria sprayed on the tomato leaves against some insect parasites, such as aphids, mites, and Tuta absoluta? Could be used also as insecticides?

If know, what is the effect of these selected bacteria against plant parasitic nematodes? Could be used also as nematocides?

If know, what is the impact of these selected bacteria on soil microorganisms such as collembola, mites and other micro arthropods?

*Our work was aimed at the evaluation of these selected bacteria against fungal pathogens. Actually, we don’t have results against other organisms but future studies could investigate the activity of selected bacteria against other target organisms.*

Line 22 and lines 278, 295, 296: at the beginning of sentence report the entire name of the genus. *Bacillus* and not the abbreviation. *Starkeya* and not the abbreviation. Check it in all the MS.

*All suggested changes are now included in the text.*

Lines 58-60: I suggest to insert e.g. after the first bracket and to delete etc at the end

*Suggested changes are now included in the text.*

Line 84: I suggest to change with culturing the isolates.

*In our opinion, the sentence is correct and do not needs this change.*

Line 93: Specify where the contaminated soil was collected.

*The contaminated soil was collected in proximity of a fuel distributor located in Campania Region (Italy).*

Lines 149-157: Where is the control? It is not clear what bacterial strains were used. Please, clarify.

*The text was modified according to your suggestions.*

Lines 159-171: Where is the control? It is not clear what bacterial strains were used. Please, clarify.

*The text was modified according to your suggestions.*

Lines 186-196: Where is the control? It is not clear what bacterial strains were used. Please, clarify.

*The text was modified according to your suggestions.*

Line 434-436: …screening steps through a multidisciplinary approach to obtain…

*This sentence was modified according to your suggestion.*
